# EHMTI-0321. Restricted diffusion in painful ophthalmoplegia

**DOI:** 10.1186/1129-2377-15-S1-D8

**Published:** 2014-09-18

**Authors:** JH Choi, IS Moon, SH Kim, KD Choi

**Affiliations:** 1 Neurology, Pusan National University Yangsan Hospital, Yangsan, Korea; 2 Neurology, Daedong Hospital, Busan, Korea; 3 Neurology, Dong-A University Hospital, Busan, Korea; 4 Neurology, Pusan National University Hospital, Busan, Korea

## Introduction and aims

Superior ophthalmic vein (SOV) thrombosis is a rare disease entity characterized by rapidly progressive orbital symptoms, including periorbital edema, chemosis, proptosis and painful ophthalmoplegia. Since diagnostic delays represent a life-threatening danger, early recognition of disease is important for effective treatment and good prognosis. We report a patient with SOV thrombosis showing restricted diffusion within the vein on diffusion-weighted images (DWI) which may be helpful in the diagnosis of SOV thrombosis.

## Methods and results

A 53-years-old man presented with headache and left eye swelling for 6 days. Neurological examination revealed proptosis, eyelid edema, and depression paresis in the left eye. Left pupil was dilated and sluggishly reacting to light. Orbit MRI showed diffuse enhancement in the left retro-orbital and periorbital area, and an intraluminal filling defect within the left SOV. DWI disclosed high signal intensity in the left SOV, confirmed on apparent diffusion coefficient (ADC) map by the low signal intensity in the corresponding region. These restricted diffusions were attributed to the presence of thrombus within the left SOV. Based on clinical symptoms and MRI findings, a diagnosis of SOV thrombosis caused by orbital cellulitis was made, and intravenous broad-spectrum antibiotics infusion was initiated. Over the next 7 days he showed a marked improvement of periorbital swelling, and limitation of the eye movement gradually improved.

## Conclusions

DWI may provide a clue for the presence of intravascular clots according to the stage of thrombus formation which can help the diagnostic process in SOV thrombosis.

No conflict of interest.

